# Misjudgment of Skills in Clinical Examination Increases in Medical Students Due to a Shift to Exclusively Online Studies during the COVID-19 Pandemic

**DOI:** 10.3390/jpm12050781

**Published:** 2022-05-12

**Authors:** Axel Lechner, Stefan P. Haider, Benedikt Paul, Pablo F. F. Escrihuela Branz, Axelle Felicio-Briegel, Magdalena Widmann, Johanna Huber, Ursula Stadlberger, Martin Canis, Florian Schrötzlmair, Kariem Sharaf

**Affiliations:** 1 Department of Otolaryngology, University Hospital, LMU Munich, Marchioninistrasse 15, 81377 Munich, Germany; axel.lechner@med.uni-muenchen.de (A.L.); stefan.haider@med.uni-muenchen.de (S.P.H.); benedikt.paul@med.uni-muenchen.de (B.P.); pablo.escrihuela@med.uni-muenchen.de (P.F.F.E.B.); axelle.felicio@med.uni-muenchen.de (A.F.-B.); magdalena.widmann@med.uni-muenchen.de (M.W.); martin.canis@med.uni-muenchen.de (M.C.); florian.schroetzlmair@med.uni-muenchen.de (F.S.); 2 Department of Radiation Oncology, University Hospital, LMU Munich, Marchioninistrasse 15, 81377 Munich, Germany; 3 Institute for Medical Education, University Hospital, LMU Munich, Pettenkoferstrasse 8a, 80336 Munich, Germany; johanna.huber@med.uni-muenchen.de (J.H.); ursula.stadlberger@med.uni-muenchen.de (U.S.)

**Keywords:** teaching of skills, COVID-19 pandemic, psychomotor ability, medical education, otorhinolaryngology

## Abstract

In medical school, practical capacity building is a central goal. During the COVID-19 pandemic, a shift to online teaching methods in university was mandated in many countries to reduce risk of SARS-CoV-2 transmission. This severely affected the teaching of psychomotor ability skills such as head and neck examination skills, resulting in a share of students that have only been taught such ENT-specific examination skills with online courses; our study aimed to measure performance and capacity of self-evaluation in these students. After completing a new extensive online Ear Nose Throat (ENT) examination course, we conducted a standardized clinical skills exam for nine different ENT examination items with 31 students. Using Likert scales, self-evaluation was based on questionnaires right before the clinical skills exam and objective evaluation during the exam was assessed following a standardized regime. Self-evaluation and objective evaluation were correlated. To compare the exclusive online teaching to traditional hands-on training, a historic cohort with 91 students was used. Objective examination performance after in-classroom or online teaching varied for single examination items while overall assessment remained comparable. Overall, self-evaluation did not differ significantly after online-only and in-classroom ENT skill teaching. Nevertheless, misjudgment of one’s skill level increased after online-only training compared to in-classroom teaching. Highest levels of overestimation were observed after online training in simple tasks. While gender and interest in ENT did not influence self-evaluation and misjudgment, higher age of participants was associated with an overestimation of skills. Medical students with online-only training during the COVID-19 pandemic achieved similar ENT examination skills to those with traditional on-campus training before the pandemic. Nevertheless, students with online-only training were more prone to misjudge their skills when they assessed their skills. Due to the COVID-19 pandemic, current medical students and graduates might therefore lack individual specific psychomotor skills such as the ENT examination, underlining the importance of presence-based teaching.

## 1. Introduction

Perpetual video calls and e-learning have become emblematic of the challenges brought upon university education by the COVID-19 pandemic since its onset in 2020. Colleges and universities in general, and medical schools in particular, were forced to quickly adapt curricula to ever changing social distancing provisions. These restrictions caused especially profound effects on hands-on clinical skills training. Otorhinolaryngology is among the most affected specialties, given that aerosols are generated during various clinical examinations and procedures regularly performed by ENT physicians, posing an increased risk for viral transmission [1].

With on-campus training suspended at large, and thus, limited hands-on courses and rotations offered across specialties, many medical schools resorted to online-only training [2,3,4,5,6,7,8,9,10,11,12,13,14,15,16,17,18,19,20]. Faculties and educators rushed to compile virtual curricula or to transform their curricula into e-learning programs, including clinical skills courses in ENT [2,3,5,6,7,8,9,10,12,13,14,15,16,17,18,19,20]. However, the effectiveness of such training in inducing adequate skill levels in medical students compared to traditional practical training or blended learning has not been fully established yet.

With regard to typical clinical ENT examinations, a prior study indicated that repetitive execution of intricate examinations under the supervision of experienced physicians over a prolonged period may effectively promote psychomotor skills development in medical students [21]. These findings suggest direct feedback from supervisors and hands-on training are keys to success; it remains unknown whether e-learning provides an adequate substitute in this context.

Entertaining an accurate picture of one’s own skills and expertise is crucial for clinical decision making and obviates both hubristic and hesitant actions which may equally put patients at risk. A study conducted by our group demonstrated medical students were capable of relatively accurate self-assessment of ENT examination skills after traditional hands-on skills training [22] whereas students’ ability to self-assess after online-only training has not been studied before in this setting to the best of our knowledge.

Therefore, we aimed to investigate the level of competence and psychomotor skills as well as the accuracy of self-assessment in medical students after online-training of ENT examination skills, and to compare the aforementioned cohort with one that took a blended learning skills training course developed before the onset of the pandemic.

## 2. Materials and Methods

### 2.1. Curricular Concept of Teaching

Until the winter term 2019/2020 (ending in March 2020), ENT examination skills were taught in a traditional hands-on fashion during the 4th or 5th year of the German 6-year medical school curriculum (Medical Curriculum Munich, MeCuM, LMU University) as described before [22].

We were going to introduce a new blended learning concept starting in April 2020 with the introduction of new online courses adjusted to the curricular hands-on sessions but due to the emerging COVID-19 pandemic, all hands-on sessions were suspended in the summer term of 2020, restricting skills training to the new online courses. In the winter term of 2020/2021, corresponding to a lower COVID-19 incidence rate in Germany, all aspects of the ENT examination including examination of the upper airways were allowed during one-day block courses after negative PCR testing. Furthermore, there was a mandatory clinical exam at the end of the one-day block course. Later on, these mandatory clinical exams were suspended in the summer term 2021 because of frequently changing local hygienic regulations.

To enroll students, inclusion criteria were: (a) Students were about to participate (but had not yet participated) in the one-day ENT block course, (b) needed to prepare for a mandatory clinical exam at the end of the one-day course, (c) had worked through both of our online courses, (d) voluntarily filled in the self-evaluation questionnaire, and (e) voluntarily participated in an ad hoc objective structured clinical exam (OSCE) at the very beginning of the one-day ENT block course.

In the winter term 2020/2021, there were approximately 70 to 100 students fulfilling the inclusion criteria (a) and (b). At the beginning of the one-day ENT block course upon questioning inclusion criteria (c) and (d), approximately 20 students stated that they had not yet worked through both of our online courses. Later, a further 6 students had to be excluded because they had stated in our questionnaire that they had not yet worked through both of our online courses, therefore not fulfilling inclusion criterium (c). In total, 31 students met all inclusion criteria.

### 2.2. Concept of the Online Courses

**Figure 1:**
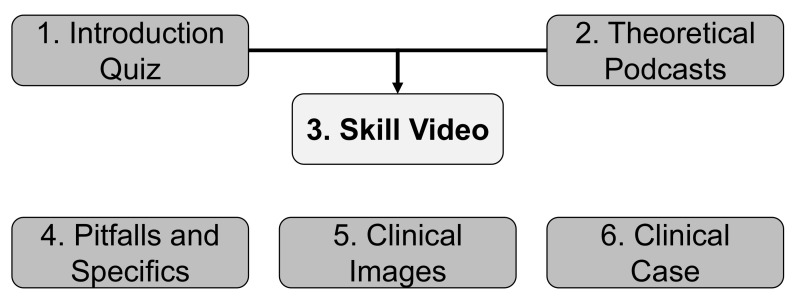
Didactic structure of an organ chapter in the online courses. To teach organ-specific examination techniques, the chapters are composed with similar didactic elements. Initially, an introduction quiz (1) is supposed to activate and self-assess pre-existing knowledge on instruments and anatomy/physiology, afterwards depending on a student‘s demand, theoretical podcasts (2) on anatomy/physiology as well as an instrument gallery are provided and leads to the central video (3) presenting organ-centered examination skills. Thereafter, galleries on pitfalls and specifics of the examination technique (4), a gallery presenting clinical images (5), and a clinical case (6) utilizing the presented examination techniques are supposed to deepen the background and understanding of the organ-specific examination.

A simplified table of contents for the basic and advanced course is shown in Table 1. The didactic conceptualization of an organ-specific chapter is depicted in Figure 1.

### 2.3. Self-Evaluation Questionnaire and Objective Structured Clinical Exam

At the very beginning of the one-day ENT block course, students were asked if they fulfilled the inclusion criteria for the study as described before and if they were willing to voluntarily participate in the study. If so, they completed a paper-based questionnaire (36 items including 13 open items) assessing basic biographic data (age, sex, number of semesters), data on the use of our online courses (e.g., duration of use for specific course elements) and self-assessed levels of nine specific ENT examination skills (otoscopy with and without microscope, tuning fork tests Weber and Rinne, anterior rhinoscopy, nasal endoscopy, examination of oral cavity and oropharynx, laryngoscopy, cervical lymph node examination, examination in case of head and neck trauma) using Likert-scales (ranging from 1 defined as ‘high proficiency’ to 5 defined as ‘low proficiency’). To perform the clinical exam, students received a written instruction for every examination item testing for theoretical knowledge of the nine specific ENT examinations their practical execution. Using the same Likert-scales, theoretical, practical, and overall skill levels were assessed by one of four experienced medical teachers in a standardized manner as performed and described before [22].

Realization of this study was based on the approval by the ethics committee of the local medical faculty (Ethikkommission der Medizinischen Fakultät der Ludwig-Maximilians-Universität, IRB approval number 19-333) and in compliance with the WMA Declaration of Helsinki.

### 2.4. Data and Statistical Analysis

We evaluated self-assessment and objective data and compared them to each other. We calculated the differences of the self-assessment and objective data of the respective examination skill with positive values resembling students that overestimated their skills and negative values resembling students that underestimated their skills. When the difference was zero, self-assessed and objective skill level matched. Because both over- and underestimation might be harmful for the future work as a medical doctor, we also determined the absolute amount of misjudgment.

To compare the extent of deviation from self- and objective examination skills and misjudgment under different teaching conditions, we used data from a historic cohort partially presented before by our group [22].

Statistical analysis was performed with GraphPad Prism 8.4.3 (San Diego, CA, USA). ANOVA was used for comparison > 2 mean values while (un-)paired group comparisons of 2 groups were calculated by Student’s *t*-test. Graphs were generated with GraphPad Prism as well.

## 3. Results

**Figure 2:**
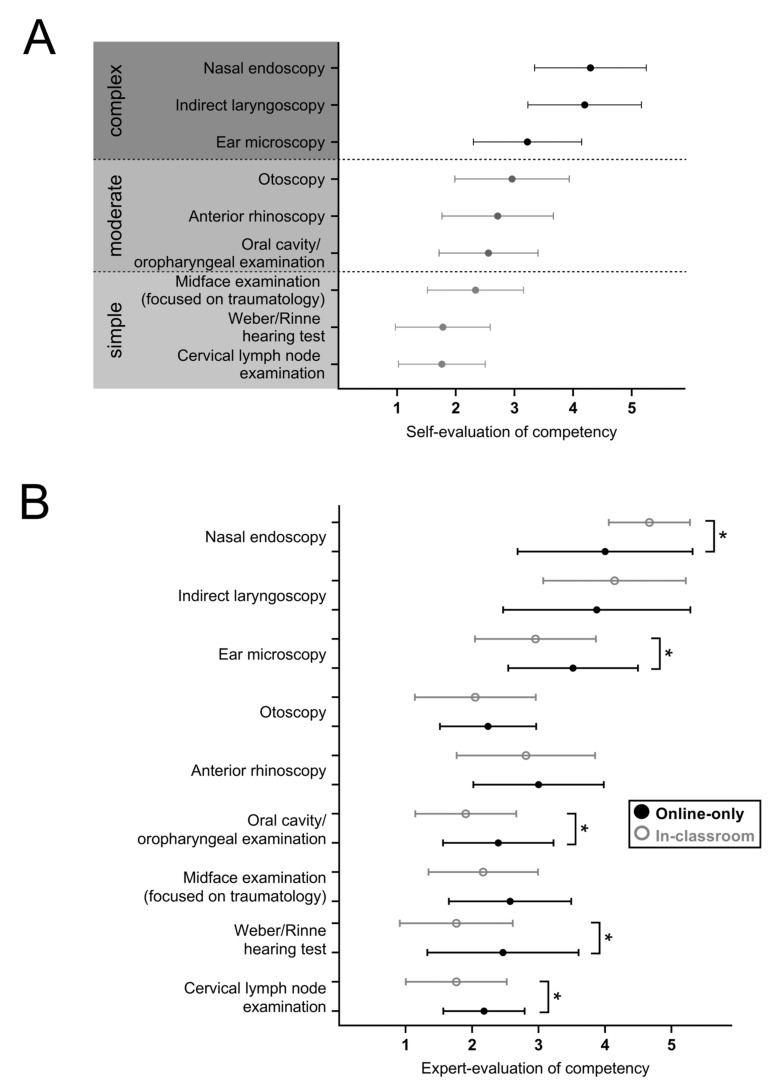
Overall self-evaluation of examination competency and comparison of expert-assessment of examination skills. (**A**) Self-evaluation of skill competency for 9 different ENT examination items is shown. Data are based on all participants irrespective of previous mode of training. Examination items are grouped into simple, moderate and complex tasks. (**B**) Expert-evaluated ratings of competencies in different examination items is shown, comparing participants who received in-classroom-teaching vs. online-only teaching. Self-/expert-evaluation is measured on a 5-point interval scale (Likert scale; 1 = high proficiency to 5 = low proficiency); * *p* < 0.05.

Characteristics of participating students are summarized in Table 2. The nine different examination items were grouped into three levels of difficulty reflecting their complexity, required fine motor skills and use of instruments. Levels were labelled as ‘simple’, ‘moderate’, and ‘complex’ (Figure 2A). Students’ self-assessment of their own skill level was consistent with the respective level of difficulty of the nine examination items (Figure 2A).

### 3.1. Overall Self-Evaluation of Examination Competency Does Not Differ Significantly after Online-Only and In-Classroom ENT Skill Teaching

At first, we investigated whether students rated their own ENT examination skill level differently after online-only or in-classroom training. Self-assessment was evaluated on a five-point interval scale (Likert scale; 1 = high proficiency to 5 = low proficiency). The overall self-assessment after in-classroom and online-only teaching was comparable (2.86 vs. 2.90, *p* = ns). Both groups considered their general physical examination skills (2.13 vs. 2.13, *p* = ns) as better than their ENT examination skills. However, students in the online-only training cohort considered profound ENT examination skills as significantly more relevant for their own clinical practice (1.84 vs. 2.20, *p* = 0.042). Self-assessment in skill level ‘simple’, ‘moderate’, and ‘complex’ did not differ significantly between both groups. However, students with online-only training rated their own skill level regarding the examination of the midface focusing on traumatology significantly better (2.07 vs. 2.43, *p* = 0.032) and anterior rhinoscopy and microscopic ear examination significantly worse compared to students after in-classroom teaching (3.03 vs. 2.60, *p* = 0.030 and 3.55 vs. 3.11, *p* = 0.043; Figure 2A).

### 3.2. Objective Examination Performance of Students Varies after In-Classroom and Online Teaching in Single Examination Items While Overall Assessment Remains Comparable

In order to compare the actual, objectively evaluated skills, the level of competency (global rating of single items) in both groups was measured on a five-point interval scale consistent with the scale that students used for assessing their own competency. The overall objective competency was rated as 2.87 in the online only group compared to 2.70 in the in-classroom cohort (*p* = ns). Thus, students in both cohorts demonstrated reasonable levels of overall ENT examination competency. While results of four single items were comparable, significant differences between both cohorts were observed in the following investigated items: performance of students after in-classroom teaching was significantly better in cervical lymph node examination (1.76 vs. 2.18, *p* = 0.018), oral/oropharyngeal examination (1.91 vs. 2.39, *p* = 0.014) and Weber and Rinne hearing tests (1.76 vs. 2.46, *p* = 0.0043). Their competency was evaluated as significantly worse in nasal endoscopy (4.67 vs. 4.00, *p* = 0.011) and microscopic ear examination (3.52 vs. 2.95, *p* = 0.017). However, the level of competency regarding these items was below their own individual expectations in both groups. Their respective level of competency remained insufficient in indirect laryngoscopy as well (4.14 vs. 3.88, *p* = ns). Results are summarized in Figure 2B.

### 3.3. Misjudgment of One’s Skill Level Increases after Online-Only Training Compared to in-Classroom Teaching

**Figure 3:**
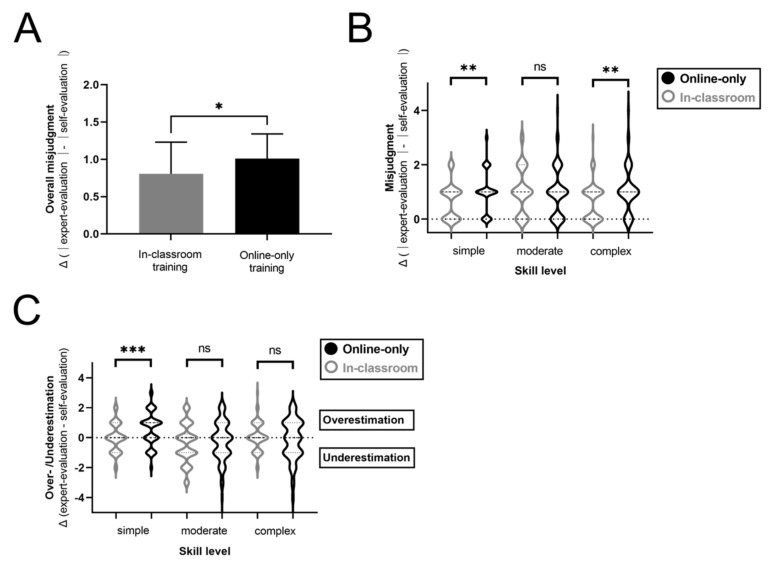
Comparison of overall misjudgment and over-/underestimation of own skills after in-classroom vs. online-only teaching. (**A**) Global overall misjudgment calculated as the difference of expert-evaluation and self-evaluation of skills irrespective of direction of potential misjudgment is compared in in-classroom vs. online-only cohorts. One global value per participant was used. (**B**) Misjudgment levels as calculated under (**A**) are shown, subdivided into respective skill levels. (**C**) Detailed analysis of over-/underestimation in different skill levels is depicted (overestimation > 0, underestimation < 0). * *p* < 0.05; ** *p* < 0.005; *** *p* < 0.0005; ns *p* > 0.05 (not significant).

Previous results focused on comparisons between both groups which did not allow for individual comparisons of self-assessment and expert evaluation. Since it is difficult to rate one’s actual skill level based on theoretical knowledge of the practical task, we subsequently investigated the level of misjudgment of students after online-only vs. in-classroom training. The discrepancy between objective rating and self-rating was used as a measure for misjudgment (over- or underestimation of own skill levels). The absolute level of misjudgment was significantly higher after online training compared to in classroom teaching (1.01 vs. 0.81, *p* = 0.023; calculated as difference between objective and self-rating irrespective of direction of misjudgment; Figure 3A). This finding could be attributed to significantly higher levels of misjudgment in simple and complex tasks (1.01 vs. 0.69, *p* = 0.0016 and 1.07 vs. 0.67, *p* = 0.0010). However, the highest levels of misjudgment overall were observed in moderate tasks (1.00 vs. 1.06, *p* = ns; Figure 3B).

### 3.4. Highest Levels of Overestimation Are Observed after Online Training in Simple Tasks

**Figure 4:**
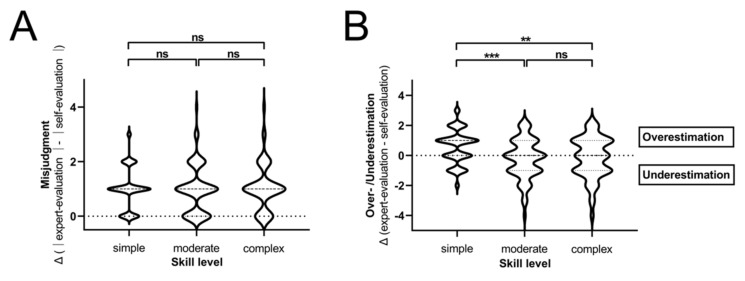
Comparison of global misjudgment and over-/underestimation in online-only cohort. (**A**) Results of misjudgment calculation in the online-only cohort are represented as violin plots subdivided into skill levels. (**B**) Levels of over- and underestimation in different skill levels in the online-only cohort are shown. ** *p* < 0.005; *** *p* < 0.0005; ns *p* > 0.05 (not significant).

Furthermore, the direction of misjudgment (overestimation or underestimation of one’s own skills) was investigated. Increased levels of overestimation of their skills were the reason for the difference between online-only and in-classroom training in simple tasks, while the level of over- and underestimation increased equally in complex tasks (Figure 3C). When comparing the three different skills levels in the online-only cohort in detail, students overestimated their own skills significantly more often in simple tasks compared to moderate and complex tasks (∆[expert rating − self-evaluation]: ∆_simple_ = 0.57 vs. ∆_moderate_ = −0.24 vs. ∆_complex_ = −0,16; Figure 4A). Accordingly, 55.6% of participants overestimated their skills in simple tasks, 29.1% in moderate and 36.8% in complex tasks. Underestimation of own skills was observed in 19.8% of students in simple, 40.5% of moderate and 40.4% of complex tasks. 24.7%, 30.4% and 36.8% matched the objective evaluation of skills in the subgroups, respectively (Figure 4B).

### 3.5. While Gender and Interest in ENT Do Not Influence Self-Evaluation and Misjudgment, Higher Age of Participants Is Associated with an Overestimation of Skills

**Figure 5:**
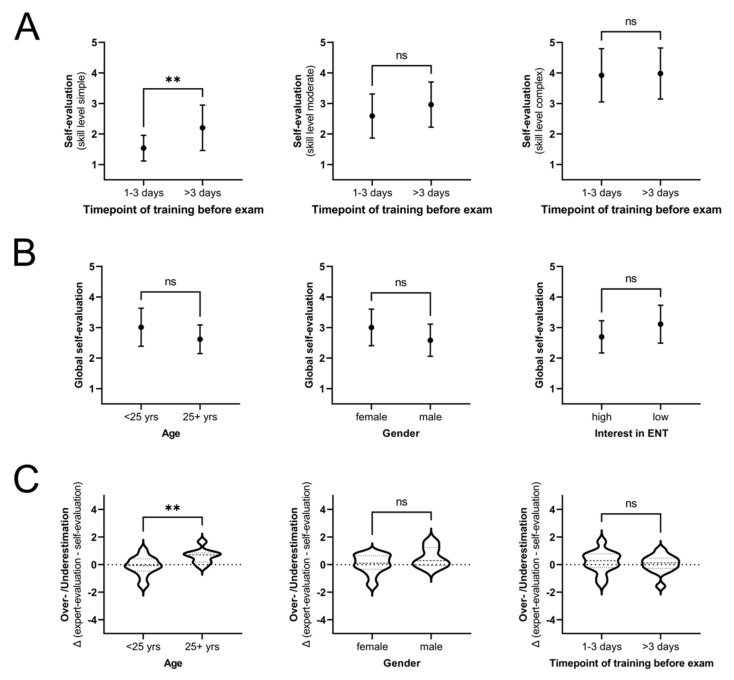
Comparison of self-evaluation and over/-underestimation in different subgroups. (**A**) Self-evaluation in different skill levels; timepoint of completion of the online training was used to define subgroups. (**B**) Global self-evaluation in different subgroups according to age, gender and interest in ENT is shown. (**C**) Global over- and underestimation is compared in age groups < 25 years and 25+ years, female vs. male and in subgroups according to completion of online training. ** *p* < 0.005; ns *p* > 0.05 (not significant).

To gain insights into potential factors that could explain these findings, subgroup analyses according to age, gender, interest in ENT, and timepoint of training were performed. Participants who did the online training within 3 days prior to the exam rated their own skills in simple tasks significantly better than students who concluded the online training more than 3 days before the test (self-evaluation 1.54 vs. 2.20; *p* = 0.007), while their actual exam performance did not differ significantly. This could not be observed in moderate and complex tasks (Figure 5A). Overall, female participants self-rated their skills tendentially worse than male participants. However, this difference was not significant (Figure 5B) and their examination performance was comparable as well. Likewise, differences of global self-evaluation according to age and interest in ENT were not significant (Figure 5B). Students younger than the median age of 25 years in the online cohort showed lower levels of misjudgment compared to the 25+ years cohort, overestimation being significantly more frequent in the latter cohort (∆ [expert rating − self-evaluation]: overall ∆_<25yrs_ = −0.11 vs. ∆_25+yrs_ = 0.63; *p* = 0.009; Figure 5C).

### 3.6. What Is the Student’s Opinion on Online Training?

Students were asked for their opinion regarding online training before the clinical exam (questionnaire with a five-point scale, 1 = fully agree to 5 = completely disagree). While the majority did not think that online training could replace practical teaching (4.56 ± 0.57), some stated that online training provides them with sufficient knowledge regarding practical ENT skills (3.60 ± 0.93) and that it is enough to prepare for a clinical exam (3.1 ± 0.92). The vast majority of students considered the online course an adequate preparation for potential in-classroom teaching sessions (1.57 ± 0.63) and a good motivation for further skills teaching (1.50 ± 0.51).

## 4. Discussion

This study aimed to investigate the potential of an exclusively online training to teach medical students ENT examination skills. Due to the COVID-19 pandemic, a significant share of medical students had to face reductions or total cancelation of on-site medical school courses on short notice, including both lectures, seminars, and hands-on courses. Frequently, curricula were changed from on-campus to exclusively online and hybrid digital teaching formats were introduced [3,4,5,9,10,13,15,20,23]. In 2019, we had drafted apposite online courses to prepare medical students how to perform ENT examinations and to deepen their examination skills in on-campus courses. Hereby, we aimed to transform our skills training from a traditional to a blended learning concept, supposedly starting in March 2020. Facing strict pandemic regulations, our online courses temporarily were the only kind of ENT skills teaching which could be offered. The results of this study reflect the examination skills and self-assessment accuracy of medical students who have used the online courses as their only curricular teaching of ENT examination skills.

Overall, the objective evaluations of the students’ examination skills showed comparative results between students who had only online course teaching and those who completed the traditional on-campus hands-on training [22]. Overall, the online-exclusive cohort and the historic on-campus cohort were comparable and their performance was almost equal, although interest in ENT was higher in the online-exclusive cohort. The gender ratio differed; however, the ratio of the recent online-exclusive cohort is more reflecting current gender ratios in German medical schools and, eventually, no gender-specific differences were found within the cohorts.

Nevertheless, we think that future students will excel these skill levels when pandemic regulations are omitted and the blended learning concept can be realized as initially planned, combining the complementary contents of the online courses with three 90 min repetitive on-site courses. Both recent studies focusing on the mandatory changes due to the pandemic regulations and other evaluated blended learning concepts have shown exemplary success in medical teaching of practical skills [2,12,24,25,26,27,28,29]. Polk et al. demonstrated that repetitive teaching produces a rapid learning curve for ENT examination skills during a one-week hands-on course [21].

During pandemic regulations, Krauss et al. evaluated a different kind of online-only training for ENT examination skills using daily video conferences during an one-week ENT block course and found that students achieved high levels of competence in ENT examination skills [30].

Following the recommendations from the meta-analysis of Blanch-Hartigan, we display self-assessment in paired comparison to the correlating objective standardized clinical examination [31]. Looking into details, we found that although students achieved similar overall and technique-specific skill levels with both a traditional and an online-only training. It stands out that the deviations from the students’ self-assessment increased with online-only training showing higher rates of both overestimation and underestimation of their examination skills. This finding is concerning as it has been hypothesized before that both overestimation and underestimation of skills in medical students can lead to false diagnostic or therapeutic conclusions and eventually endanger patients [31,32]. In our study, the comparisons of individual objective evaluation of medical teachers and subjective self-assessment of students and a broad variety of different examination skills provide robust insights in this special cohort during COVID-19 pandemic. Still, a certain weakness of this study is the relatively small sample size of the online-only cohort.

Analyzing potential influencing variables within our online cohort, we found that a higher age (25 years and older) was correlated with an overestimation of skills whereas gender and interest in ENT were not correlated with more over- or underestimation of skills. A possible explanation may be found within the German medical school admission system. A share of these older students is entering medical school from a waiting time quota resulting in a considerable extent of students who had already gained medical experiences, e.g., through a nursing staff training. Maybe, these older students deduct from their general medical experience a greater knowledge in ENT-specific items as well. These findings emphasize that medical teachers should also have a focus on specifics of the student cohort such as age.

Overall, we conclude that online-only teaching for ENT examination techniques is a good alternative option in exceptional circumstances such as the COVID-19 pandemic but should not generally replace on-campus skill training. Moreover, our findings suggest that medical teachers and curriculum planners as well as residency program directors need to recognize and adapt to the fact that the current generation of medical students and graduates might lack individual skills due to the direct or indirect effects of the COVID-19 pandemic.

Even before the COVID-19 pandemic, studies had shown that medical graduates do not feel prepared for several different skills that one would expect from a medical graduate [33,34]. In a recent study, Canadian medical school graduates stated that a majority of students had minimal exposure to ENT during medical school and most of them have low confidence managing ENT conditions [35]. Review courses and obligatory curricular tests of skills such as United States Medical Licensing Examination (USMLE) Step 2 Clinical Skills that obliges medical students to review and train a broad spectrum of clinical skills could certainly help to reduce these individual deficiencies.

## 5. Conclusions

This exploratory study revealed that medical students with online-only training during the COVID-19 pandemic regulations achieved similar ENT examination skills as those with a traditional on-campus training before the pandemic. Nevertheless, students with online-only training were more likely to both overestimate and underestimate their skills when they were asked to assess themselves. Medical school curricula and residency programs should acknowledge that current medical students and graduates, that had to face reduced skills training during medical school due to the COVID-19 pandemic, might lack individual specific psychomotor skills such as the ENT examination.

## Figures and Tables

**Table 1:** Simplified course content of the new ToSkORL (Teaching of Skills in Otorhinolaryngology) online courses.

Chapter	Basic Course	Advanced Course
**Otology**	Key skill: Examination of the ear using an otoscope Supplementary skills: Subjective audiometry using a tune fork (Weber and Rinne testing)	Key skill: Examination of the ear using an ear microscope Supplementary skills: Ear wax/foreign body removal; subjective and objective audiometry
**Rhinology**	Key skill: Anterior rhinoscopy using a nasal speculum Supplementary skills: bipolar coagulation of nosebleed	Key skill: Endoscopy of the nose and sinuses Supplementary skills: percussion of the sinuses and cranial nerve testing
**Laryngology/Stomatology**	Key skill: Examination of oral cavity and oropharynx using spatula Supplementary skills: Examination of the salivary glands	Key skill: Examination of hypopharynx and larynx using 70° endoscopes Supplementary skills: Transnasal flexible laryngoscopy
**Head and Neck**	Key skill: Examination of the cervical lymph nodes and swellings Supplementary skills: Examination of the thyroid gland	Key skill: Trauma examination of the head and neck Supplementary skills: Examination of different cranial nerves
**Occupational interest**	Introduction into the history of Otorhinolaryngology	Educational aspects to become and occupational profile of an Otorhinolaryngologist

**Table 2:** Cohort characteristics.

		Online-Only Teaching	In-Classroom Teaching
Number of students		*n* = 31	*n* = 91
Age ^1^		25 ± 3.1 years	26 ± 4.1 years
Gender ^2^	Female	23 (74.2%)	46 (50.5%)
	Male	8 (25.8%)	44 (48.4%)
	n.a.	0 (0.0%)	1 (1.1%)
Semester ^1^		8.9 ± 0.7	8.9 ± 0.6
Interest in ENT (1 = very high to 5 = very low) ^3^		2.55 ± 0.85	2.92 ± 0.88
Self-assessment of general physical examination skills (1 = high proficiency to 5 = low proficiency) ^1^		2.13 ± 0.76	2.13 ± 0.88
Timepoint of online course completion	1–3 days prior to exam	13 (41.9%)	
	4–7 days prior to exam	6 (19.4%)	
	>7 days prior to exam	11 (35.5%)	
	n.a.	1 (3.2%)	

Comparison of the cohorts’ characteristics showed significant differences regarding gender and interest in ENT (^2^ *p* = 0.035 Two-sided Fischer’s exact test; ^3^ *p* = 0.042 unpaired *t*-test) and no significant differences regarding age, semester, and self-assessment of general physical examination skills (^1^ age *p* = 0.228, semester *p* = 0.917, self-assessment of general physical examination skills *p* = 0.484; unpaired *t*-test). Background color: in-classroom cohort did not have the online course, not applicable.

## Data Availability

Data and corresponding statistical analyses of this study are available on request from the corresponding authors.
